# Supplementary material for: Biomimetic Nanopillar Silicon Surfaces Rupture Fungal Spores
Source: Int J Mol Sci. 2023 Jan 9;24(2):1298. doi: 10.3390/ijms24021298 (PMC9864238; doi:10.3390/ijms24021298)
Supplement: Supplementary file 1 [file ijms-24-01298-s001.zip › ijms-2109912-supplementary.pdf]

# Supplementary Information

# Biomimetic Nanopillar Silicon Surfaces Rupture Fungal Spores

**Denver P. Linklater [1,2,†], Phuc H. Le [1,2,†], Arturo Aburto-Medina [1,2], Russell J. Crawford [1], Shane Maclaughlin [2,3], Saulius Juodkazis [4] and Elena P. Ivanova [1,2,*]**

[1] School of Science, STEM College, RMIT University, Melbourne, VIC 3000, Australia; denver.linklater@rmit.edu.au (D.P.L.); s3756681@student.rmit.edu.au (P.H.L.); arturoaburto.medina@rmit.edu.au (A.A.-M.); russell.crawford@rmit.edu.au (R.J.C.)

[2] ARC Research Hub for Australian Steel Manufacturing, Wollongong, NSW 2505, Australia; shane.maclaughlin@bluescopesteel.com

[3] BlueScope Steel Ltd., Port Kembla, Wollongong, NSW 2505, Australia

[4] Optical Science Centre, Swinburne University of Technology, Hawthorn, Melbourne, VIC 3122, Australia; sjuodkazis@swin.edu.au

* Correspondence: elena.ivanova@rmit.edu.au

† These authors contributed equally to this work.

# Supplementary text

## 1. Materials and Methods

### 1.1. Plate counting technique

After a 1-day incubation period, the surfaces were washed with 1.5 mL MilliQ water by pipetting gently 5 × 3 min to remove non-attached conidia. The surfaces were then vortexed in an Eppendorf tube and a portion of 100 µL was collected for plate counting. Serial dilutions were performed and 100 µL of diluted suspension (Dilution factor: $10^{-2}$) were inoculated and spread to each PDA plate to obtain colony forming units (CFU $mL^{-1}$). Plates were incubated at room temperature in the dark for 72 h, then the colonies were counted. For statistical analysis, three independent replicates were performed and there were at least three agar plates for each type of surface.

### 1.2. Metabolic assay

The samples were immersed in *A. brasiliensis* suspension for a 7-day incubation period in a 24-well plate (corning). Cell respiration was assessed on day 3 and day 7. At each incubation point, 10% v/v pre-mixed alamarBlue™ (Thermo Fisher Scientific, USA) was added to each well containing surfaces with attached conidia and incubated for 3 h. Then, the fluorescence intensity (Excitation 530-560/ Emission 590) was measured by using a CLARIOstar plate reader (BMG Labtech, Mornington, Australia).

**Supplementary Figures**

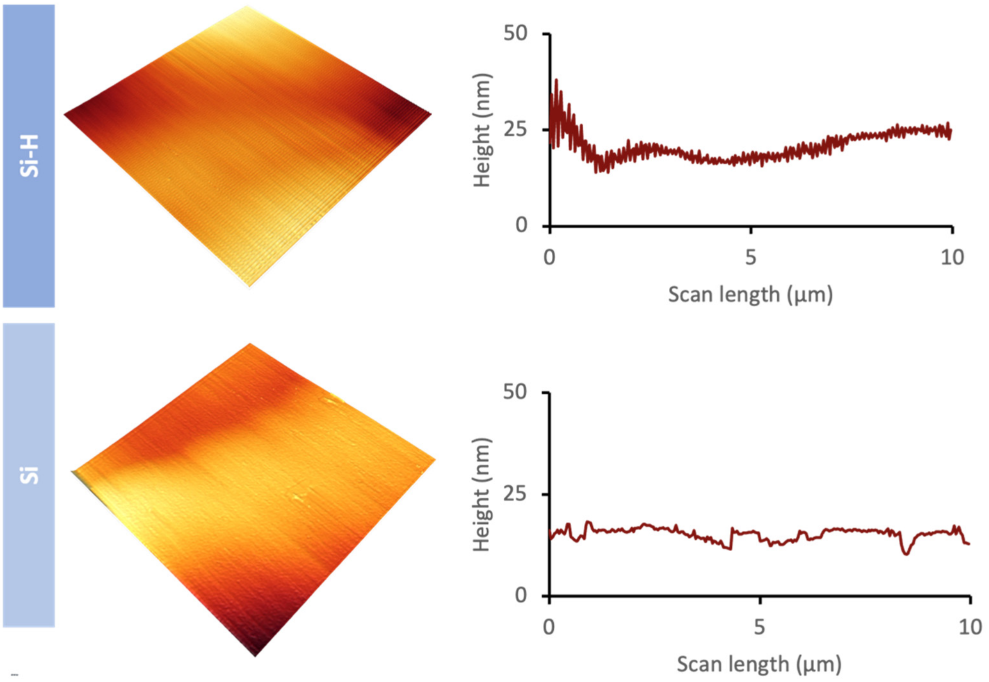


**Figure S1**. Surface topography analysis of Si-H and Si surfaces. Corresponding 3D visualisation of AFM micrographs and AFM line profiles demonstrated the surface topography of Si-H and Si surfaces.

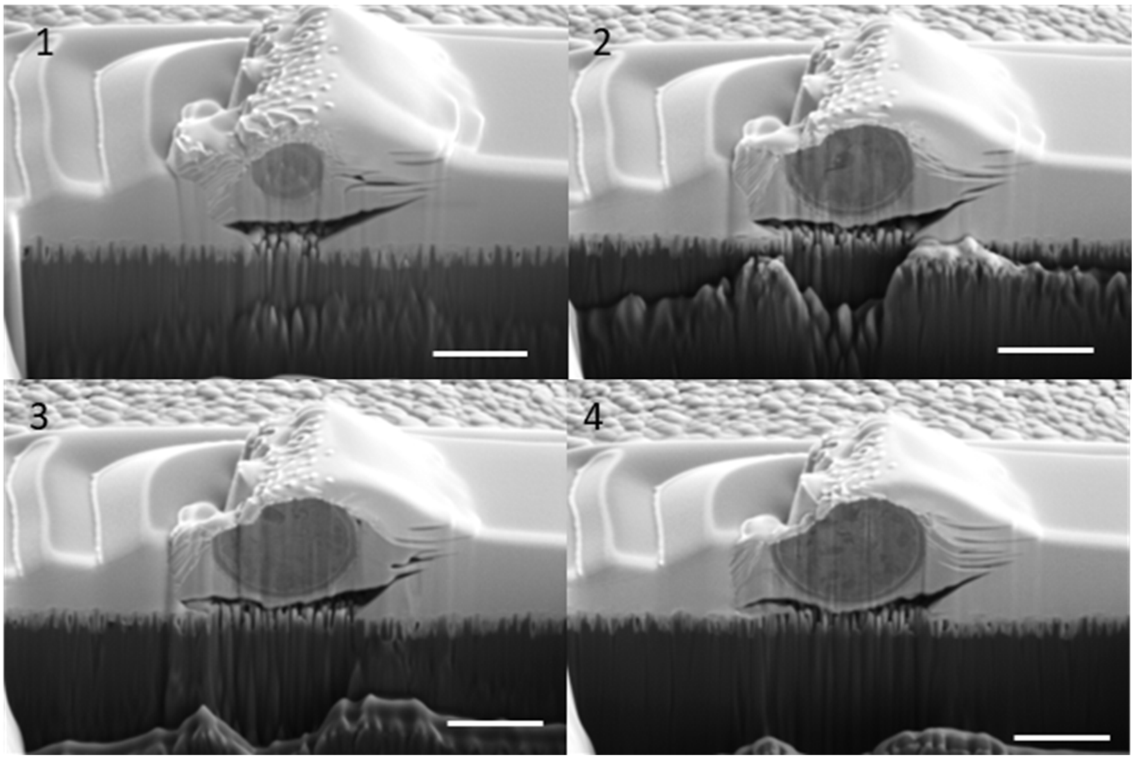


**Figure S2**. SEM micrographs of consecutive FIB-milling of the spore-nanopillar interface on nSi-H surfaces. Scale bars are 2 μm.

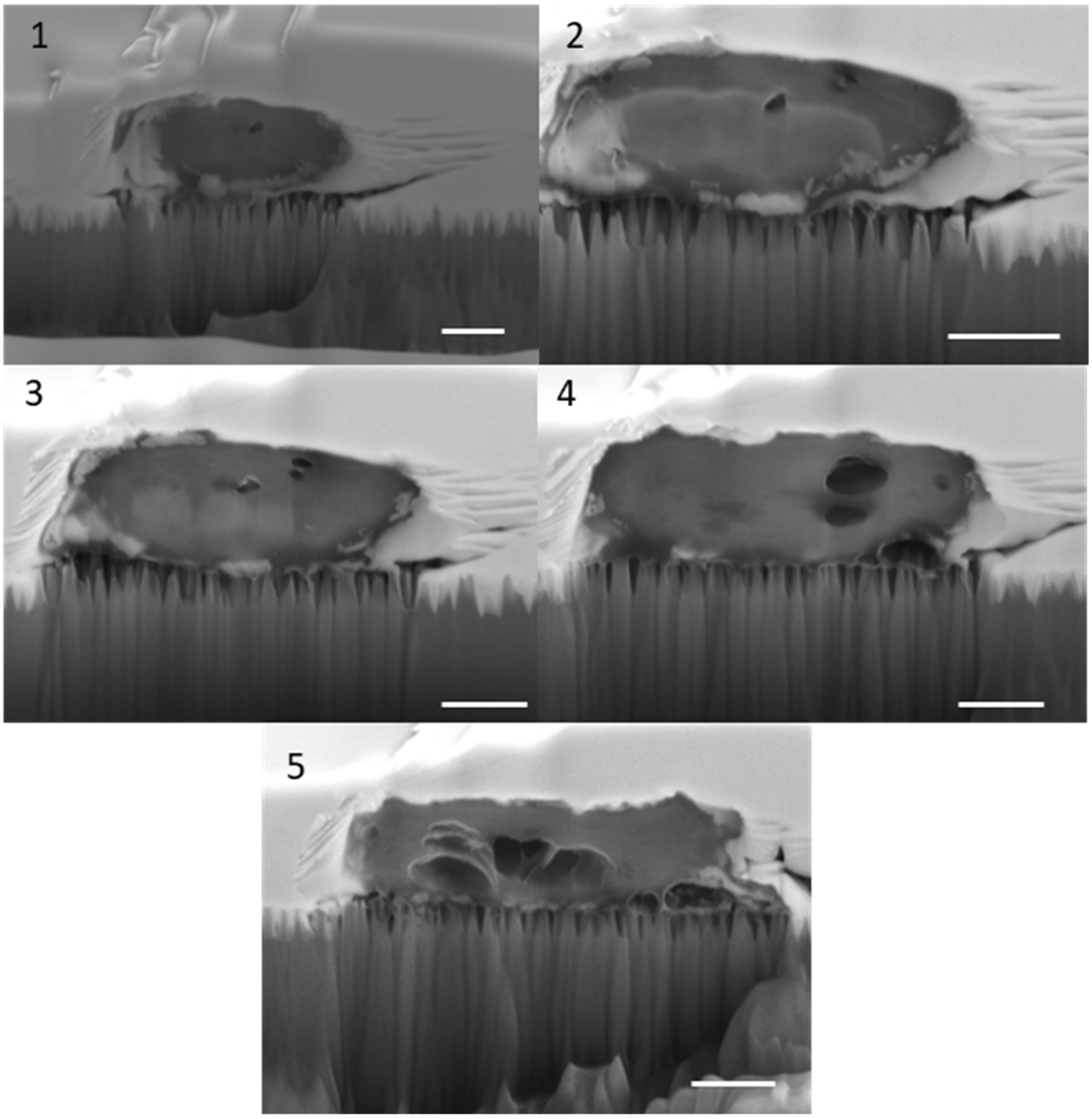


**Figure S3**. SEM micrographs of consecutive FIB-milling of the spore-nanopillar interface on nSi surfaces. Scale bars are 1 μm.

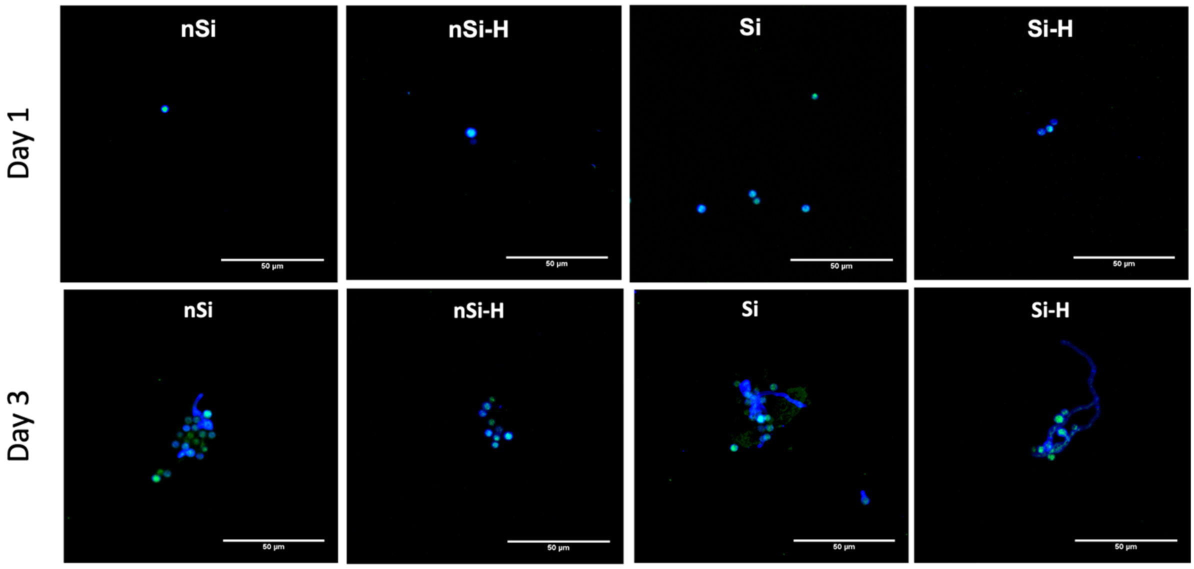


**Figure S4**. Representative CLSM micrographs of the attachment of *A. brasiliensis* spores on studied surfaces over 1-day and 3-day incubation period. The fungal spore coats were stained with Calcofluor white (blue colour) and the nuclei with NucSpot (green colour). *A. brasiliensis* spores formed long hyphae and developed in large communities after a 3-day incubation period on Si-H and Si surfaces, respectively. Scale bars are 50 µm.

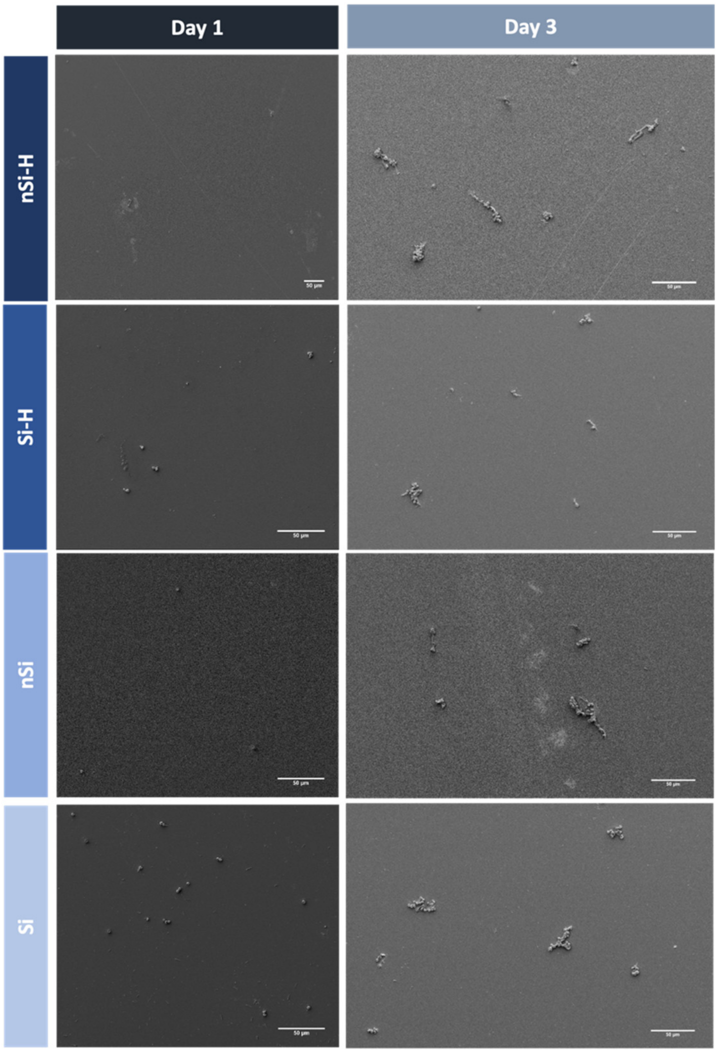


**Figure S5**. The attachment of *A. brasiliensis* spores to bSi-H, Si-H, bSi and Si surfaces over 3-day incubation period, where the surfaces were immersed in the conidial suspension.

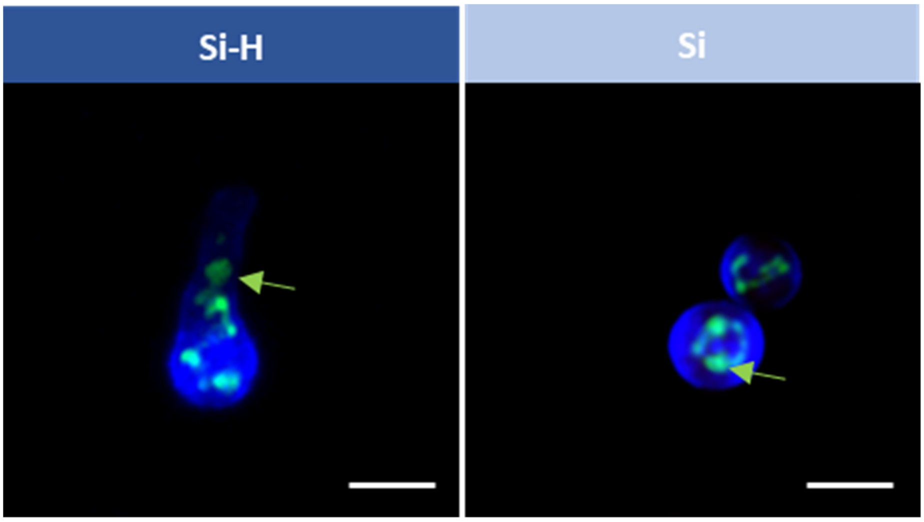


**Figure S6**. Representative CLSM micrographs of spores attached on Si-H and Si planar surfaces. No PI uptake was detected. Scale bars are 5 µm.

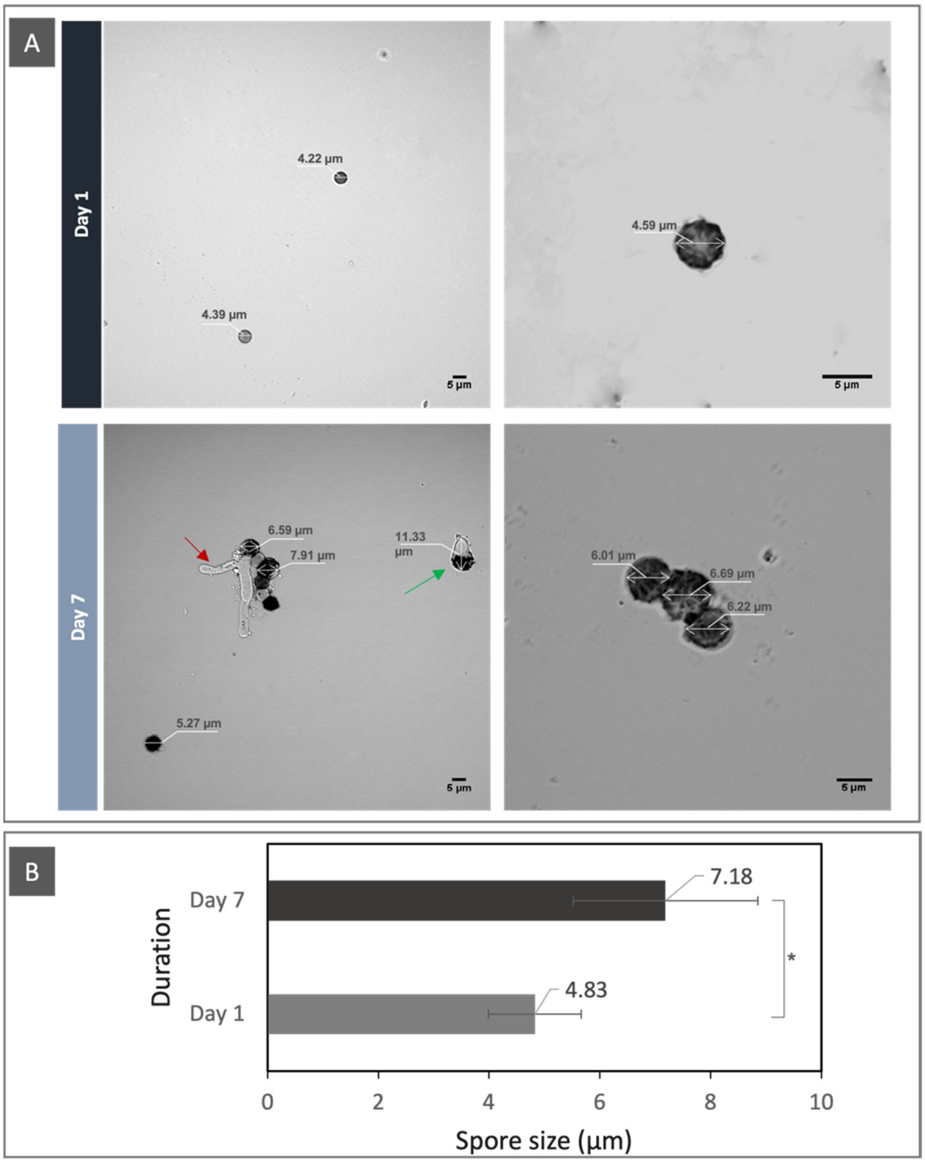


**Figure S7.** *A. brasiliensis* spore sizes after a 7-day period incubation in aqueous conditions. (A) The CLSM micrographs of *A. brasiliensis* spores taken under bright field mode. The initial density of spore suspension was $10^6$ per mL. The spore size of non-germinated spores was approximately 4.83 µm, increasing approximate 1.5 time in diameter after 7 days incubating in water. After 7 days, the *A. brasiliensis* spores also showed the germination process, including germinating (green arrow) and forming hyphae (red arrow). (B) The average spore sizes (in diameter) of non-germinated and germinated *A. brasiliensis spores* after 1 and 7-day incubated in water, respectively. The asterisk indicates the statistically significant difference in the spore size between Day 1 and Day 7, n = 50 (*$p < 0.05$).
